# SARS-CoV-2 Vaccination-Induced Immunogenicity in Heart Transplant Recipients

**DOI:** 10.3389/ti.2023.10883

**Published:** 2023-02-06

**Authors:** Felix Memenga, Simon Thomas Kueppers, Katrin Borof, Paulus Kirchhof, Paul Maria Duengelhoef, Markus Johannes Barten, Marc Lütgehetmann, Filip Berisha, Nina Fluschnik, Peter Moritz Becher, Christoph Kondziella, Alexander M. Bernhardt, Hermann Reichenspurner, Stefan Blankenberg, Christina Magnussen, Meike Rybczynski

**Affiliations:** ^1^ Department of Cardiology, University Heart and Vascular Center, University Medical Center Hamburg-Eppendorf, Hamburg, Germany; ^2^ German Center for Cardiovascular Research (DZHK), Partner Site Hamburg/Kiel/Lübeck, Hamburg, Germany; ^3^ Institute of Immunology, University Medical Center Hamburg-Eppendorf, Hamburg, Germany; ^4^ Department of Cardiovascular Surgery, University Heart and Vascular Center, University Medical Center Hamburg-Eppendorf, Hamburg, Germany; ^5^ Institute of Medical Microbiology, Virology and Hygiene, University Medical Center Hamburg-Eppendorf, Hamburg, Germany; ^6^ German Center for Infection Research (DZIF), Partner Site Hamburg/Lübeck/Borstel/Riems, Hamburg, Germany

**Keywords:** immunosuppression, heart transplantation, humoral response, COVID-19 vaccination, T-cell response

## Abstract

Among heart transplant (HT) recipients, a reduced immunological response to SARS-CoV-2 vaccination has been reported. We aimed to assess the humoral and T-cell response to SARS-CoV-2 vaccination in HT recipients to understand determinants of immunogenicity. HT recipients were prospectively enrolled from January 2021 until March 2022. Anti-SARS-CoV-2-Spike IgG levels were quantified after two and three doses of a SARS-CoV-2 vaccine (BNT162b2, mRNA1273, or AZD1222). Spike-specific T-cell responses were assessed using flow cytometry. Ninety-one patients were included in the study (69% male, median age 55 years, median time from HT to first vaccination 6.1 years). Seroconversion rates were 34% after two and 63% after three doses. Older patient age (*p* = 0.003) and shorter time since HT (*p* = 0.001) were associated with lower antibody concentrations after three vaccinations. There were no associations between vaccine types or immunosuppressive regimens and humoral response, except for prednisolone, which was predictive of a reduced response after two (*p* = 0.001), but not after three doses (*p* = 0.434). A T-cell response was observed in 50% after two and in 74% after three doses. Despite three vaccine doses, a large proportion of HT recipients exhibits a reduced immune response. Additional strategies are desirable to improve vaccine immunogenicity in this vulnerable group of patients.

## Graphical Abstract

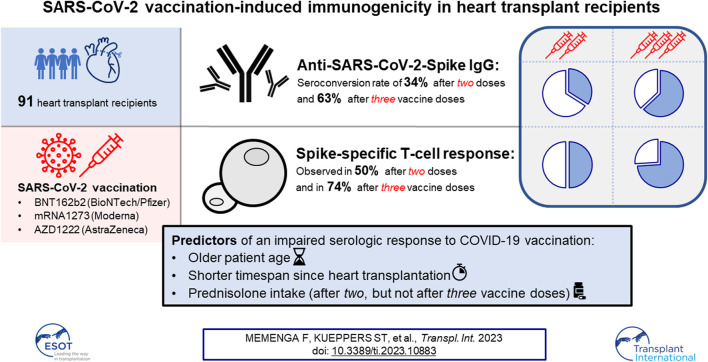

## Introduction

The clinical management of heart transplant (HT) recipients during the ongoing COVID-19 (coronavirus disease 2019) pandemic has been challenging, as these patients are at high risk of severe clinical impairment and adverse outcomes upon infection with severe acute respiratory syndrome coronavirus-2 (SARS-CoV-2) (1–3). Several vaccines with high efficacy against SARS-CoV-2 infection and good safety profiles have been approved, including the mRNA-based vaccines BNT162b2 (Tozinameran, Pfizer-BioNTech, New York City, USA/Mainz, Germany) and mRNA1273 (Spikevax, Moderna, Cambridge, USA), and the non-replicating viral vector vaccine AZD1222 (Vaxzevria, AstraZeneca, Cambridge, United Kingdom) (4–6). Vaccination of HT recipients has been recommended by the International Society for Heart and Lung Transplantation (ISHLT) (7). However, as immunocompromised individuals have been largely excluded from clinical trials, there is a paucity of data on the immunological response after vaccination of solid organ transplant (SOT) recipients.

Recent studies have reported a reduced humoral response to SARS-CoV-2 vaccination in SOT recipients (8–13), who may have an especially low probability for seroconversion after two vaccine doses compared to other immunocompromised patients (13). Seroconversion rates in HT recipients after two doses vary widely in the current literature (from 10% to 75%) (9–12,14). To improve vaccine responses, the ISHLT currently recommends three doses of an mRNA vaccine as primary series (15). Additional booster doses have been proposed and modification of immunosuppressive regimens are being investigated in ongoing trials (16,17). Impaired humoral responses have been associated with older patient age, shorter time since transplantation, and immunosuppression with anti-metabolite agents such as mycophenolate mofetil (9,10,14). In addition to circulating antibodies, T-cell activity is an important component of the immune response against SARS-CoV-2 infection (18,19). So far, only few studies have analyzed T-cell immunity in vaccinated HT recipients (14,20,21).

Here, we report quantification of the humoral and T-cell response after a second and third dose of a COVID-19 vaccine in a consecutive cohort of heart transplant patients seen at a large transplant center. We also report determinants of vaccine response in this cohort.

## Patients and Methods

### Study Participants and Data Collection

From January 2021 until March 2022, we enrolled HT recipients that presented to the HT outpatient clinic of the University Heart & Vascular Center Hamburg, a large tertiary care center. Clinical variables including age, sex, date of transplantation, immunosuppressive medications, renal function *via* estimated glomerular filtration rate (eGFR) and history of diabetes were assessed at time of registration.

**FIGURE 1:**
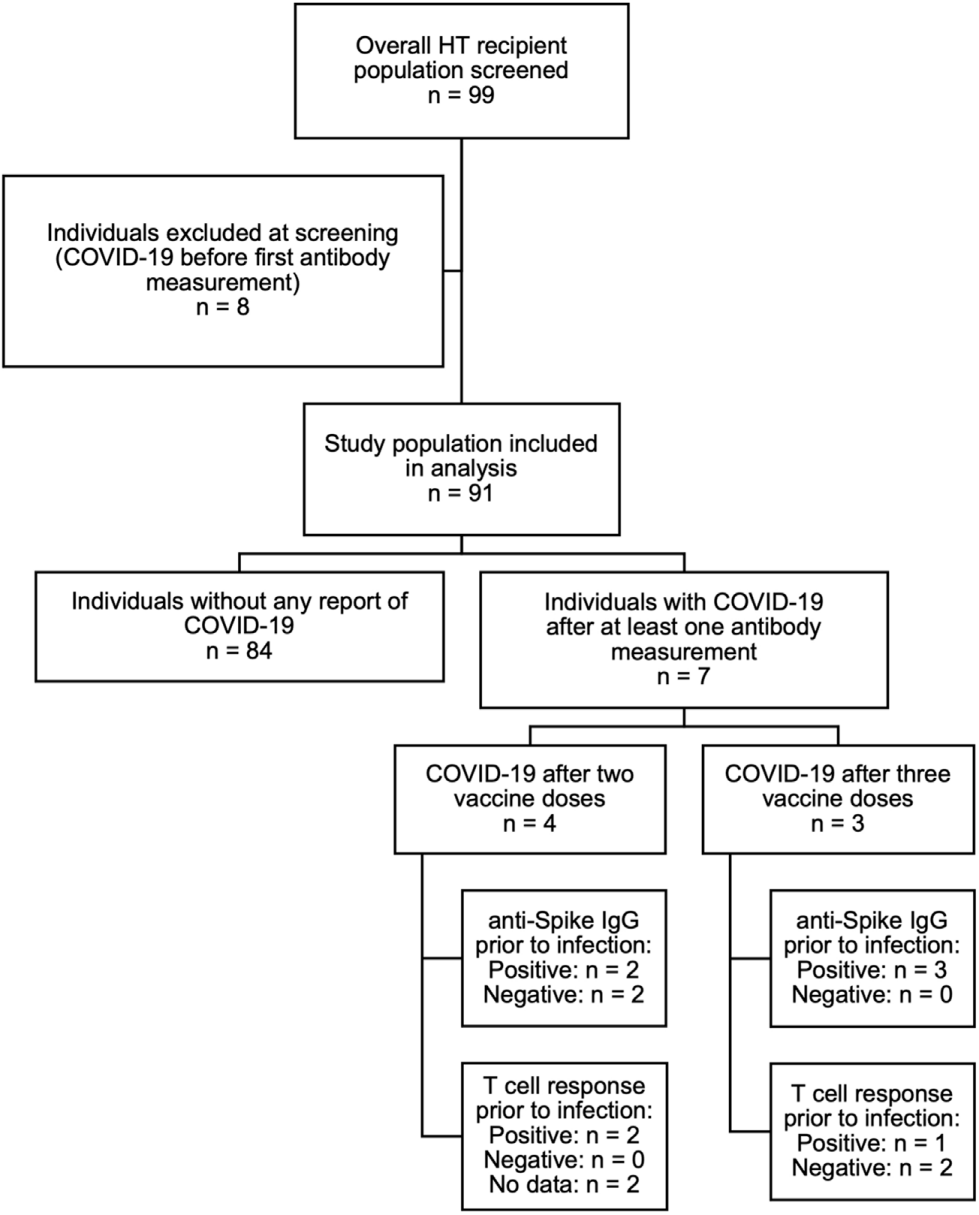
Overview of the study population.

Participants had previously received two doses of the mRNA-based vaccines BNT162b2 (Pfizer-BioNTech) and mRNA-1273 (Moderna), or the viral vector-based AZD1222 (AstraZeneca) vaccine. After each the second and the third vaccination, anti-SARS-CoV-2 IgG concentrations in the blood serum and, in a subset of patients, spike-specific T-cell responses were assessed during routine ambulatory follow-up visits. Vaccinations had been administered by the patients’ primary care physicians or by specialized vaccination centers in accordance with the German prioritization guidelines and recommendations of the standing vaccination committee (STIKO) (22). We did not include any HT recipients with a known history of COVID-19 prior to the first sampling timepoint. Also, if a participant developed COVID-19 after the first samples were taken, all measurements obtained after infection were excluded from the analysis (Figure 1). Accordingly, we did not include any measurements after administration of therapeutic monoclonal antibodies against SARS-CoV-2 in our analysis. Rates of COVID-19 infection during the study period were low, with only 7 cases reported after at least one antibody measurement. The study was approved by the local ethics committee (PV 6079) and conducted in concordance with the Declaration of Helsinki. Written informed consent was provided by all participants.

### Assessment of SARS-CoV-2 Vaccine-specific Humoral and T-cell Response

We assessed the vaccine-specific humoral response after a median of 42 days (interquartile range [IQR] 29.0–98.8) after the second vaccination (“pre-booster”) and 39.5 days (28.0–62.0) after the third vaccination (“post-booster”). The DiaSorin LIAISON XL anti‐SARS‐CoV‐2 TrimericS IgG ChemiLuminescent ImmunoAssay (sensitivity 99.4%, specificity 99.8%) (23) was used to quantitatively determine the anti-SARS-CoV-2-Spike IgG (anti-S Trimer) levels. As proposed by the manufacturer, positive humoral response was defined by an anti-S Trimer IgG concentration of ≥33.8 BAU/mL (23).

The spike-specific T-cell response was assessed using an activation-induced marker assay (AIM) similar to previous studies (24,25). In detail, peripheral blood mononuclear cells were isolated from EDTA‐blood *via* density gradient centrifugation (Lymphocytes Separation Media, Capricorn Scientific, Ebsdorfergrund, Germany) and frozen at −80°C. After thawing, a minimum of 1 × 10^6^ cells were stimulated with an overlapping 15‐mer peptide pool derived from the full sequence of the SARS‐CoV‐2 spike glycoprotein (PepMix^TM^ SARS‐CoV‐2 Spike Glycoprotein, JPT Peptide Technologies, Berlin, Germany) or left unstimulated for 18 h at 37°C after adding 1 μL Ultra‐LEAF^TM^ purified anti‐human CD40 antibody (BioLegend, San Diego, USA). Cells were stained with antibody-mix for the detection of surface molecules (see Supplementary Table S1 for antibodies used). All samples were analyzed on a BD FACS Canto II, and FlowJo version 10.8.0 (BD Biosciences, Franklin Lakes, USA) was used for the flow cytometric analysis (see Supplementary Figure S1 for gating strategy).

In accordance with a previous study on T-cell immunity after SARS-CoV-2 vaccination using a similar assay (25), a positive T-cell response was defined by a stimulation index (SI) ≥2 calculated by dividing CD154^+^CD137^+^CD4^+^ T-cells in the stimulated samples by the corresponding cells in the unstimulated samples. SIs below 1 were set to 1.

### Statistical Analysis

Continuous variables are presented as median with interquartile range (25th percentile to 75th percentile), and categorical variables as absolute numbers (relative frequencies). Pearson’s Chi-squared test, the Wilcoxon rank sum test and Fisher’s exact test were used to investigate the effect of the type of vaccine, vaccination regimen, and immunosuppressive agents on the immune response.

We analysed the association of several non-modifiable characteristics with antibody levels and seroconversion rates, namely patient age at first vaccine dose, sex, the timespan between vaccination and serological measurements, and timespan from HT to the first vaccination, and performed multivariable analyses (logistic and Tobit regression analyses) to identify determinants of seroconversion. Further, we used a Tobit regression model to account for values below the limit of detection of the assay used (<4.81 BAU/mL, which affected *n* = 34 after two and *n* = 21 after three vaccine doses). The Tobit model is a special case of the more general censored regression model and is designed to estimate linear relationships between variables when there is either left- or right-censoring in the dependent continuous variable (26,27).

Antibody concentrations were log-transformed for linear and Tobit regression analysis. For logistic regression models, we used the manufacturer’s threshold for antibody positivity (≥33.8 BAU/mL) to differentiate between positive and negative antibody responses, as described above. The effect of immunosuppressive agents on seroconversion rates was assessed in multivariable logistic regression analyses adjusting for age at first vaccine dose, sex, and an interaction effect between the two. The effect of prednisolone use on IgG concentrations was also studied adjusting for the timespan from HT to vaccination (in addition to age and sex) in a Tobit linear regression model since prednisolone is often included in immunosuppressive regimens in the first years after HT.

A two-tailed *p*-value <0.05 was considered statistically significant. All calculations were made using statistical computing software R (Version 4.0.5.) (28).

## Results

### Baseline Characteristics

Of 99 patients screened, 8 patients were excluded due to a SARS-CoV-2 infection prior to the first serologic assessment, resulting in a total of 91 HT recipients to be included in the study. Sixty-three patients were male (69%) and 28 female (31%). Median age was 55 years (IQR 48.5–61) and median time from HT to first vaccination was 6.1 years (1.6–13.2). Seventy-seven patients (85%) were treated with calcineurin inhibitors (62 [68%] with tacrolimus and 15 [16%] with ciclosporin), 41 (45%) with mycophenolate, 67 (74%) with everolimus and 49 (54%) with low-dose prednisolone (generally 5 mg per day). Forty-one patients (45%) were on dual, 48 (53%) on triple, and 2 (2%) on quadruple immunosuppressive therapy. All patients on a triple therapy regimen except for one received prednisolone. The most common immunosuppressive regimen was everolimus combined with tacrolimus, with or without prednisolone (24 patients [26%] and 21 patients [23%], respectively). A history of diabetes was reported in 27 patients (30%), and median eGFR was 49.0 mL/min (IQR 34.0–69.0) (Table 1). Antibody concentrations were available in 82 participants after two and in 70 participants after three vaccine doses.

**TABLE 1:** Baseline characteristics.

	Overall, N = 91^a^	Sex		
		Female, N = 28^a^	Male, N = 63^a^	
Clinical characteristics				
Patient age at first vaccine dose [years]	55 [48.5, 61]	54 [41.8, 60]	55 [50.5, 62]	
Time from HT to first dose [years]	6.1 [1.6, 13.2]	4.3 [1.7, 10.6]	7.2 [1.5, 13.2]	
Time between first and second dose [days]	42.0 [35.0, 42.0]	42.0 [38.5, 42.2]	41.0 [35.0, 42.0]	
Time between second dose and first antibody measurement [days]	42.0 [29.0, 98.8]	37.0 [27.0, 94.8]	44.5 [32.5, 94.2]	
Time between third vaccination and second antibody measurement [days]	39.5 [28.0, 62.0]	32.5 [24.2, 75.8]	41.0 [32.8, 48.2]	
History of type 2 diabetes mellitus	27 (30%)	5 (18%)	22 (35%)	
Estimated glomerular filtration rate (eGFR [mL/min])	49.0 [34.0, 69.0]	46.5 [33.2, 62.0]	52.0 [34.0, 69.0]	
Immunosuppressive therapy				
Everolimus use	67 (74%)	22 (79%)	45 (71%)	
Cyclosporine use	15 (16%)	4 (14%)	11 (17%)	
Mycophenolate mofetil use	41 (45%)	8 (29%)	33 (52%)	
Prednisolone use	49 (54%)	16 (57%)	33 (52%)	
Tacrolimus use	62 (68%)	23 (82%)	39 (62%)	
CNI regimen				
Regimen containing CNI	77 (85%)	27 (96%)	50 (79%)	
CNI-free regimen	14 (15%)	1 (3.6%)	13 (21%)	
Drug combinations				
Tacrolimus + Everolimus + Prednisolone	24 (26%)	11 (39%)	13 (21%)	
Tacrolimus + Everolimus	21 (23%)	8 (29%)	13 (21%)	
Tacrolimus + Mycophenolate mofetil + Prednisolone	8 (8.8%)	1 (3.6%)	7 (11%)	
Tacrolimus + Mycophenolate mofetil	7 (7.7%)	2 (7.1%)	5 (7.9%)	
Tacrolimus + Everolimus + Mycophenolate mofetil + Prednisolone	2 (2.2%)	1 (3.6%)	1 (1.6%)	
Cyclosporine A + Mycophenolate mofetil + Prednisolone	5 (5.5%)	2 (7.1%)	3 (4.8%)	
Cyclosporine A + Mycophenolate mofetil	4 (4.4%)	1 (3.6%)	3 (4.8%)	
Cyclosporine A + Everolimus + Prednisolone	3 (3.3%)	1 (3.6%)	2 (3.2%)	
Cyclosporine A + Everolimus + Mycophenolate mofetil	1 (1.1%)	0	1 (1.6%)	
Cyclosporine A + Everolimus	2 (2.2%)	0	0	
Everolimus + Mycophenolate mofetil + Prednisolone	7 (7.7%)	0	7 (11%)	
Everolimus + Mycophenolate mofetil	7 (7.7%)	1 (3.6%)	6 (9.5%)	

^a^ Median [IQR] or Frequency with number (%); Missing data excluded.

Continuous variables with few values and/or few different values are shown as categorical.

HT heart transplantation; CNI calcineurin inhibitor.

### Details of SARS-CoV-2 Vaccination

**FIGURE 2:**
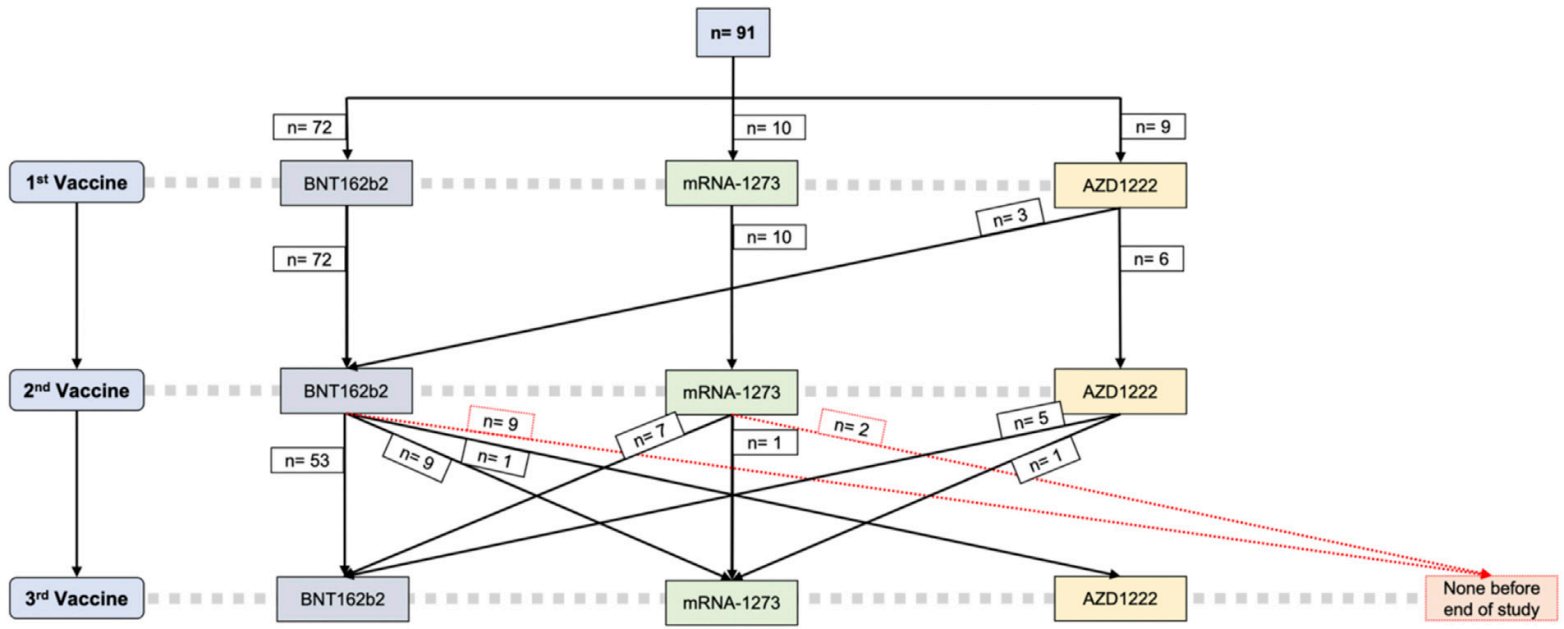
SARS-CoV-2 vaccine regimens. BNT162b2 Tozinameran, Pfizer-BioNTech; mRNA-1273 Spikevax, Moderna; AZD1222 Vaxzevria, AstraZeneca.

All patients screened received at least two SARS-CoV-2 vaccinations. Most participants (79%) received BNT162b2 as their first and second vaccine doses, while 11% received two doses of mRNA-1273. Of the 9 patients (9.9%) vaccinated with a first dose of AZD1222 (AstraZeneca), only 6 received a second dose of AZD1222, whereas the other 3 were switched to BNT162b2 as the second vaccine. The median time span between the two primary vaccinations was 42 days (35.0, 42.0). Regarding the third vaccination, more than two thirds (68%) received a third dose of an mRNA vaccine matching the primary vaccination (homologous vaccine regimen), while 32% were switched from BNT162b2 to mRNA-1273 or *vice versa*. Patients that had received two doses of AZD1222 received an mRNA-based vaccine as their third dose, either BNT162b2 (6.2%) or mRNA-1272 (1.2%) (Figure 2).

More than half of the patients (52%) reported no (solicited or unsolicited) vaccination-associated adverse event whatsoever. Systemic reactogenicity was reported by 29.2%, including fatigue (16%), fevers and chills (5.5%), headaches (4.4%) and myalgia (3.3%), and local reactogenicity in the form of pain at the injection site by 27%. There were no vaccine-related adverse events requiring professional medical attention in our cohort.

### Humoral and Spike-Specific T-Cell Response

**FIGURE 3:**
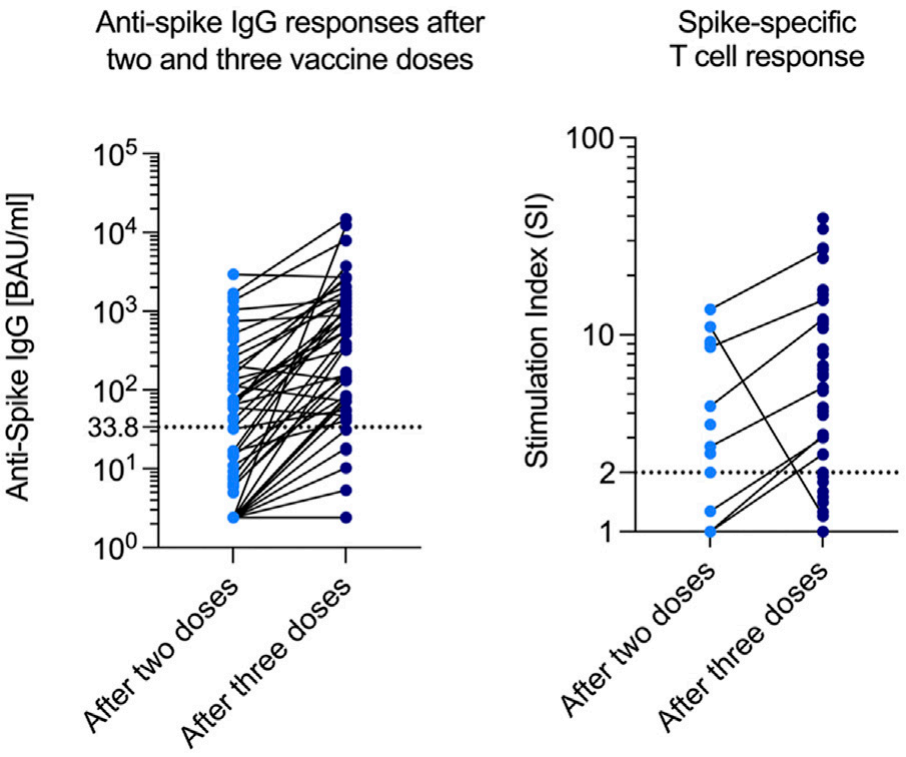
Anti-Spike IgG and SI after two and three doses. SI Stimulation index.

After two vaccine doses, a positive humoral response could be detected in 31 out of 82 patients (37.8%), and the median antibody concentration was 74.6 BAU/mL (14.9–358.0). After three doses, the median antibody concentration was 553.0 BAU/mL (80.1–1,400.0), and the number of participants with seroconversion rose to 44 out of 70 in which antibody concentrations were measured (62.9%). A third vaccine dose nearly doubled the probability of a positive humoral response (Figure 3).

The spike-specific T-cell response was measured in a subset of 49 patients: In 18 patients after two vaccine doses, and in 39 patients after three doses (data after both two and three doses were available for 8 patients). A positive T-cell response was observed in 9 out of 18 patients after two doses (50%), compared to 29 out of 39 patients (74%) after three doses. Interestingly, out of these 29 patients with a detectable T-cell response, 8 (28%) did not show a humoral response (Table 2).

**TABLE 2:** Humoral and spike-specific T-cell response for the whole study population.

	Overall, N = 91^a^	Sex		*p*-value^b^
		Female, N = 28^a^	Male, N = 63^a^	
Humoral response			
Anti-SARS-CoV-2 spike IgG after 2nd dose [BAU/mL]^c^ (*n* = 48)	74.6 [14.9, 358.0]	113.0 [45.7, 234.0]	59.2 [14.4, 473.0]	0.520
Seroconversion^d^ after 2nd dose (*n* = 82)	31/82 (38%)	14/26 (54%)	17/56 (30%)	0.041
Anti-SARS-CoV-2 spike IgG after 3rd dose [BAU/mL]^c^ (*n* = 49)	553.0 [80.1, 1,400.0]	675.0 [131.0, 1,400.0]	456.0 [77.9, 1,332.5]	0.535
Seroconversion^d^ after 3rd dose (*n* = 70)	44/70 (63%)	16/22 (73%)	28/48 (58%)	0.247
Spike-specific T-cell response			
SI after 2nd dose (n = 18)	2.2 (1.0, 7.6)	2.7 (1.1, 7.7)	2.0 (1.0, 6.1)	0.5
Positive response after 2nd dose (*n* = 18)	9/18 (50%)	4/7 (57%)	5/11 (45%)	>0.9
SI after 3rd dose (n = 39)	5 (2, 12)	6 (3, 15)	4 (2, 11)	0.3
Positive response after 3rd dose (*n* = 39)	29/39 (74%)	12/14 (86%)	17/25 (68%)	0.3

^a^ Median [IQR] or Frequency with number (%); Missing data excluded.

^b^ Wilcoxon rank sum test; Fisher’s exact test; Pearson’s Chi-squared test; Wilcoxon rank sum exact test.

^c^ Anti-SARS-CoV-2 spike IgG calculated without non-measurable patients.

^d^ Anti-SARS-CoV-2 spike IgG ≥33.8 BAU/mL.

Continuous variables with few values and/or few different values are shown as categorical.

SI, stimulation index.

### Predictors of Immunogenicity

In multivariable logistic regression analyses, higher patient age at vaccination was identified as a predictor of lower seroconversion rates (odds ratio [OR] 0.95, 95% confidence interval [CI] 0.91–0.99, *p* = 0.013), whereas a longer timespan from HT to vaccination was a predictor of higher seroconversion rates (OR 1.1, 95% CI 1.02–1.19, *p* = 0.018) after additional adjustment for patient sex. While seropositivity was observed in 14 out of 26 (54%) female and 17 out of 56 (30%) male participants (*p* = 0.041) in which data were available after two vaccine doses, this difference was not preserved after adjusting for age and timespan from last vaccination to antibody measurement in a multivariable logistical regression model (*p* = 0.085). Here, sex alone was not an independent predictor of an impaired response to vaccination. When sex was interacting with age, however, we saw a trend that middle-aged men reached a positive antibody response more frequently than women, but this effect reversed with increasing age (see Supplementary Figure S2). Above the age of 55, women may have reached a positive antibody response more frequently than men. After three doses, there was no significant sex-related difference in antibody positivity overall (*p* = 0.247) and in the same regression model (*p* = 0.321). The timespan from last vaccine dose to antibody measurement was not an independent predictor of antibody response after two (*p* = 0.132) or three doses (*p* = 0.756) after adjustment for age and sex in another logistic regression model.

Using a pre-defined threshold for severe renal impairment of an eGFR <30 mL/min, we could not detect a significant influence of eGFR on log-transformed anti-Spike IgG levels after adjusting for age and timespan from HT to vaccination in a Tobit regression model. Of note, only 13 patients in our cohort had an eGFR below this threshold. While a history of type 2 diabetes mellitus (T2DM) was reported in 27 patients (30%), the median age in this group was higher compared to non-diabetic HT recipients (60 years [IQR 54.5–63] vs. 54 years [41–60], *p* = 0.007). T2DM was not predictive of seropositivity after three vaccine doses (*p* = 0.3), even when adjusting for patient age in a logistic regression analysis.

The vaccine types, and whether a homologous or heterologous scheme was used, did not influence the humoral response to the second or third vaccine dose, respectively. These results persisted in a logistic regression analysis comparing seropositivity rates after homologous and heterologous vaccine schemes, adjusting for age and sex (OR 1.24, 95% CI 0.41–3.87, *p* = 0.707).

In a multivariable logistic regression model adjusting for age, sex and the interaction effect between both these variables, prednisolone intake was associated with lower seroconversion rates after both two (OR 0.12, 95% CI 0.03–0.38, *p* < 0.001) and three vaccine doses (OR 0.34, 95% CI 0.11–0.95, *p* = 0.046, Table 3). Similar results were seen in a Tobit linear regression model with log-transformed anti-spike IgG concentrations as the outcome variable (see Supplementary Table S3). Patients on a three-drug immunosuppressive regimen exhibited a lower seropositivity rate after two doses (OR 0.09 [0.02–0.28], *p* < 0.001 after adjusting for age and sex), while a trend towards a lower immunogenicity after the third dose remained (OR 0.37 [0.12–1.06], *p* = 0.071). In contrast, everolimus intake was associated with an increased antibody response after three (but not after two) doses in the univariable logistic regression analyses and the Tobit linear regression model (see Supplementary Tables S2, S3), but this effect did not meet statistical significance in the logistic regression model (OR 3.1 [1.01–10.01], *p* = 0.051, see Table 3). Other immunosuppressive agents, including mycophenolate, did not affect humoral responses (Table 3). After two vaccine doses, the predictive effect of prednisolone was preserved even after adjusting for the timespan from HT to vaccination (*p* = 0.001). Conversely, after three vaccine doses and adjusting for the same variables, there was no significant influence of prednisolone intake (*p* = 0.434).

**TABLE 3:** Logistic regression results for the association between the two-dose or three-dose antibody positivity outcome and exposure to immunosuppressive drug use, adjusted for age at first vaccination and sex as an interaction term.

	Antibody positivity after two vaccine doses (n = 82)													
Predictors	OR	*p*	OR	*p*	OR	*p*	OR	*p*	OR	*p*	OR	*p*	OR	*p*
Age at first vaccine dose	1.04 (0.98–1.11)	0.240	1.03 (0.97–1.10)	0.303	1.04 (0.98–1.11)	0.210	1.03 (0.97–1.10)	0.322	1.01 (0.95–1.09)	0.656	1.04 (0.98–1.11)	0.172	1.03 (0.98–1.10)	0.266
Male sex	791.10 (9.48–138251.09)	0.006	749.86 (8.31–141907.54)	0.007	958.61 (10.61–185645.85)	0.005	867.15 (8.97–175602.99)	0.006	194.23 (1.61–51378.07)	0.043	1,386.33 (14.29–292694.91)	0.004	964.21 (10.69–186659.88)	0.005
Age at first vaccine dose * Male sex	0.86 (0.78–0.94)	0.001	0.87 (0.78–0.94)	0.002	0.86 (0.78–0.94)	0.001	0.86 (0.78–0.94)	0.002	0.89 (0.80–0.97)	0.012	0.86 (0.78–0.93)	0.001	0.86 (0.78–0.94)	0.002
Everolimus use			2.05 (0.62–7.60)	0.255										
Cyclosporine A use					0.67 (0.12–3.29)	0.633								
Mycophenolate use							0.37 (0.12–1.09)	0.078						
Prednisolone use									0.12 (0.03–0.38)	<0.001				
Tacrolimus use											2.35 (0.69–9.14)	0.188		
Use of any calcineurin inhibitor													3.12 (0.59–26.35)	0.224

	Antibody positivity after three vaccine doses (n = 70)													
Predictors	OR	*p*	OR	*p*	OR	*p*	OR	*p*	OR	*p*	OR	*p*	OR	*p*
Age at first vaccine dose	1.00 (0.93–1.06)	0.913	1.01 (0.94–1.07)	0.817	0.99 (0.93–1.06)	0.870	1.00 (0.94–1.07)	0.883	0.99 (0.92–1.05)	0.707	0.99 (0.93–1.06)	0.867	1.00 (0.93–1.06)	0.920
Male sex	1.17 (0.01–101.76)	0.944	1.48 (0.01–138.00)	0.867	1.17 (0.01–103.15)	0.945	1.62 (0.01–155.78)	0.837	1.00 (0.01–103.15)	1.000	1.18 (0.01–103.84)	0.943	1.18 (0.01–102.10)	0.943
Age at first vaccine dose * Male sex	0.99 (0.91–1.07)	0.742	0.98 (0.91–1.07)	0.692	0.99 (0.91–1.07)	0.750	0.98 (0.91–1.07)	0.717	0.99 (0.91–1.08)	0.806	0.99 (0.91–1.07)	0.735	0.99 (0.91–1.07)	0.731
Everolimus use			3.10 (1.01–10.01)	0.051										
Cyclosporine A use					1.18 (0.28–5.52)	0.828								
Mycophenolate use							0.41 (0.13–1.19)	0.106						
Prednisolone use									0.34 (0.11–0.95)	0.046				
Tacrolimus use											0.82 (0.27–2.45)	0.730		
Use of any calcineurin inhibitor													0.87 (0.23–3.10)	0.832

OR, odds ratio.

Antibody positivity: anti-SARS-CoV-2 spike IgG concentration ≥33.8 BAU/mL.

Regarding the spike-specific T-cell responses, no association between either of the non-modifiable predictors mentioned above or the immunosuppressive regimen and the SI after three vaccine doses could be established.

## Discussion

This report details the humoral and cellular immune response to up to three SARS-CoV-2 vaccinations in a large, consecutive cohort of HT patients. We observed seroconversion rates of 34% and 63% and a T-cell response in 50% and 74% after two and three vaccine doses, respectively. Higher age and shorter time since transplantation were identified as predictors of seroconversion, while there was no association with vaccine type and type of immunosuppressive therapy.

While all approved SARS-CoV-2 vaccines have repeatedly and thoroughly proven to be safe in use (4–6), no serious adverse events related to vaccination were reported in our cohort. Vaccine-related effects like injection-site reactions or fever were less frequently observed compared to rates reported in these trials. Apart from safety issues, there has been ongoing debate about their immunogenicity and efficacy in immunocompromised individuals (10,11,13). The low seroconversion rates observed after two vaccine doses are in line with findings from recent studies (9,10,12), and lower than in the general population (29). Impaired vaccine responses in SOT recipients (30,31) and immunocompromised patients in general (32) have been well documented before the SARS-CoV-2 pandemic, and linked to both the primary disease-associated morbidity, the immunosuppressive medications or a combination of both. The diminished humoral response in HT recipients after SARS-CoV-2 vaccination has been shown to improve upon a third dose (20), which is strongly supported by our results. A significant subgroup of patients remains without detectable antibodies and thus in desperate need for additional strategies in terms of prevention from infection with SARS-CoV-2 and protection against severe course of the disease (13).

We identified several non-modifiable predictors for an impaired humoral response, including higher patient age, and a shorter timespan from HT to vaccination. This is consistent with previous reports (10,14). The former may be associated with a generally suboptimal antibody and T-cell response after SARS-CoV-2 vaccination among the elderly (33), while the latter may be related to a more intensive immunosuppressive therapy in the first years after HT, with most patients being on triple immunosuppressive therapy (14). In our study, prednisolone was in almost all cases used within a triple immunosuppressive regimen, so our observations regarding the association of prednisolone and 3-drug regimens with the humoral response before and after the third vaccination support the consistency of our findings, and suggest that additional booster doses may help attenuate or even overcome certain inhibitory effects of immunosuppressive agents (or triple combinations) on antibody production. Previous studies in SOT recipients reported an impaired response after two vaccine doses in patients on mycophenolate mofetil (9,11), especially when higher doses were used (32), which is not supported by our data. In contrast, our findings suggest a positive effect of everolimus use on vaccine-induced immunogenicity. However, further research is warranted, especially regarding multiple booster doses, before recommendations on adjustments or withdrawal of immunosuppressive drugs can be deduced.

**FIGURE 4:**
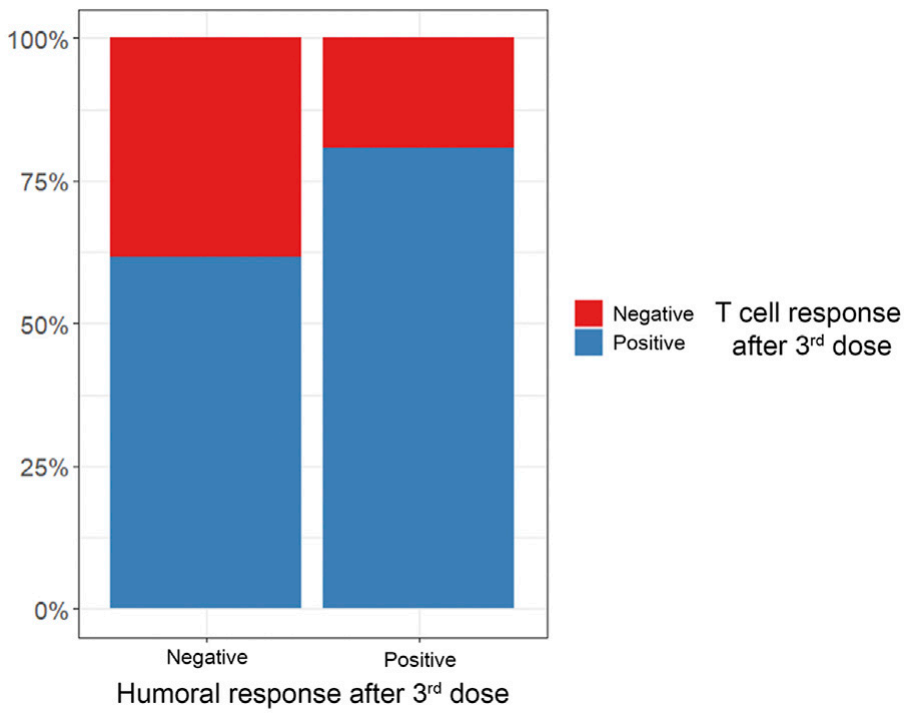
Percentage of positive vs. negative T-cell responses in relation to antibody response after 3^rd^ vaccine dose.

While antibody production represents a major mechanism of vaccine-induced immunogenicity, eliciting a T-cell response is considered important for long-term protection against severe disease after SARS-CoV-2 vaccination (34). To date, data on vaccine-induced cellular immunity in HT recipients is scarce. While overall T-cell response rates were slightly higher than antibody seroconversion rates in our cohort, 28% of patients with a positive T-cell response were seronegative after three doses (Figure 4; Table 4). This is consistent with a previous study that reported a significant proportion of HT recipients to remain seronegative after two doses although showing a positive T-cell response (21). While the clinical impact of seronegativity in light of an existing cellular response remains unclear, there is consensus on the increased vulnerability of patients without any type of response (21). SOT recipients at high risk for or suspected insufficient immunogenicity despite repeated vaccination may benefit from recently introduced pre-exposure prophylaxis (PrEP) using monoclonal antibodies (mABs) like AZD7442 (35). Further studies are needed to establish both the role of multiple additional vaccine doses in non-responders and assess the protective ability of mABs with different variants of concern currently circulating, which have not been present during phase 3 vaccine trials.

**TABLE 4:** T-cell response after three vaccine doses in relation to humoral response.

	Overall, *n* = 39^a^	Spike-specific T-cell response		*p*-value^b^
		Negative, *n* = 10^a^	Positive, *n* = 29^a^		
Antibody positivity				0.3
Negative	13 (33%)	5 (50%)	8 (28%)	
Positive	26 (67%)	5 (50%)	21 (72%)	

^a^ n (%).

^b^ Fisher’s exact test.

### Strengths and Limitations

There are several advantages inherent to the single-center nature of the data, such as the consecutive enrollment of study participants, complete data capture and the homogeneous management regimen. On the other hand, the small sample size limits statistical power and generalizability of the findings. The small case number of COVID-19 infections of participants during the study period (see Figure 1) also limits statistical analysis of the available serology and/or T-cell data prior to infection and precludes evaluation of vaccine efficacy in this cohort.

In our study, the activation-induced marker assay for the assessment of the specific T-cell response was only applied in a subset of patients, which might have influenced conclusions on the interaction between cellular and humoral immunity. While rates of reported SARS-CoV-2 infection during the study period were low in our population, we did not assess antibody levels to SARS-CoV-2 nucleocapsid protein and thus cannot exclude undetected or asymptomatic infections prior to sample acquisition.

### Conclusion

Despite ISHLT-recommended SARS-CoV-2 vaccination schedules, a significant proportion of HT recipients exhibit insufficient humoral and T-cell responses. Patient age and time since transplantation predict lower immunogenicity, but inhibitory effects of prednisolone (within 3-drug immunosuppressive regimens) on antibody production may be attenuated through booster vaccination. More data on the effect of immunosuppressive agents on immune response is warranted to improve management of this exceptionally vulnerable group of patients.

## Data Availability

The raw data supporting the conclusion of this article will be made available by the authors, without undue reservation.

## Abbreviations

AIM, Activation-induced marker assay; AZD1222, Vaxzevria (AstraZeneca, Cambridge, United Kingdom); BAU/mL, Binding antibody units per milliliter; BNT162b2, Tozinameran (Pfizer, New York City, USA; BioNTech, Mainz, Germany); CD, Cluster of differentiation; CI, Confidence interval; COVID-19, Coronavirus disease 2019; EDTA, Ethylenediaminetetraacetic acid; eGFR, Estimated glomerular filtration rate; HT, Heart transplantation; IgG, Immunoglobulin G; IQR, Interquartile range; ISHLT, International Society for Heart and Lung Transplantation; mAB, Monoclonal antibody; mRNA, Messenger ribonucleic acid; mRNA1273, Spikevax (Moderna, Cambridge, USA); OR, Odds ratio; PrEP, Pre-Exposure Prophylaxis; SARS-CoV-2, Severe acute respiratory syndrome coronavirus-2; SI, Stimulation index; SOT, Solid organ transplant; STIKO, Standing vaccination committee (Germany), “Ständige Impfkommission”; T2DM, Type 2 diabetes mellitus.
