# Supplementary material for: SARS-CoV-2 Vaccination-Induced Immunogenicity in Heart Transplant Recipients
Source: Transpl Int. 2023 Feb 6;36:10883. doi: 10.3389/ti.2023.10883 (PMC9939437; doi:10.3389/ti.2023.10883)
Supplement: Supplementary file 1 [file DataSheet1.PDF]

SARS-CoV-2 vaccination-induced immunogenicity in heart transplant recipients

Supplementary Data

Supplementary Figure 1: Gating strategy

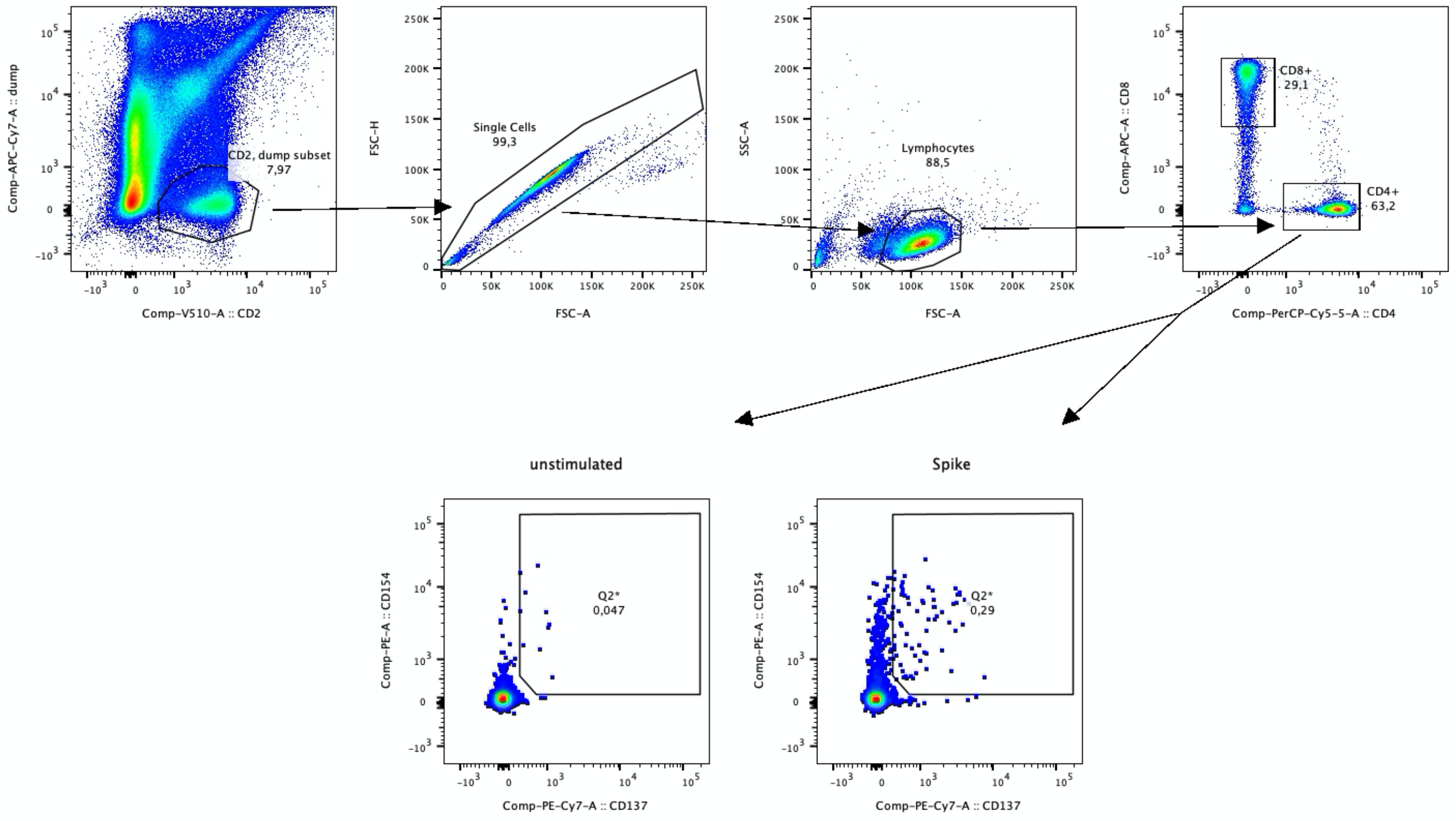

Supplementary Figure 2: Interaction of Age and Sex in a logistic regression model visualized using a probability scale

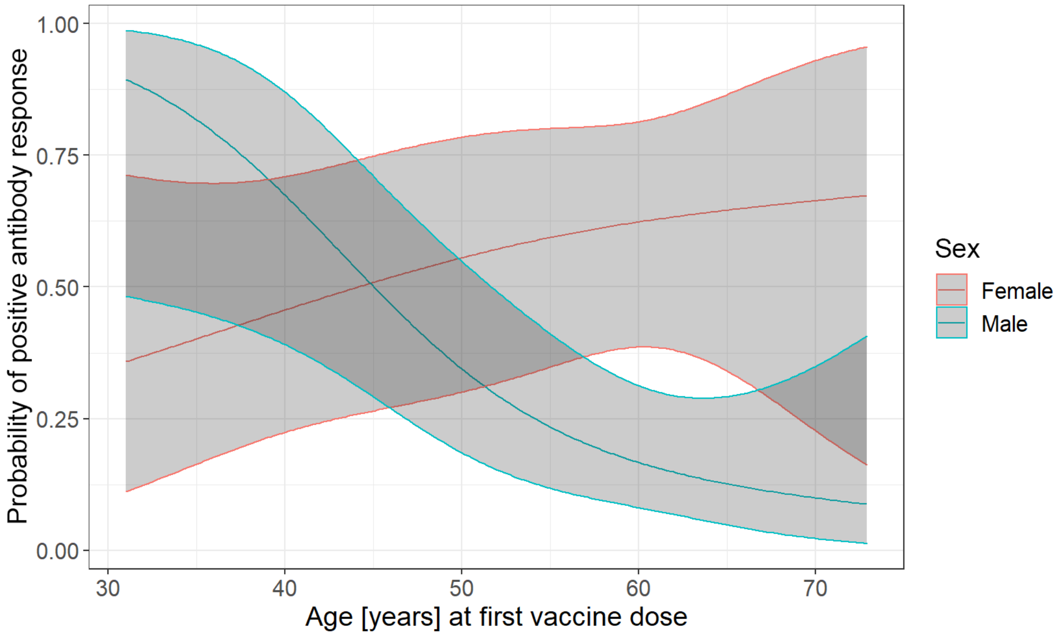

Supplementary Table 1: List of antibodies used

Antigen	Fluorophore	Cat. no.	Clone	Supplier
CD27	BV421/V450	562513	M-T271	BD Biosciences
CD2	BV510	300218	RPA-2.10	BioLegend
CD45RA	FITC/AF488	304106	HI100	BioLegend
CD154	PE	130-113-613	REA238	Miltenyi
CD4	PerCPCy5.5	300530	RPA-T4	BioLegend
CD137	Pe-Cy7	309818	4B4-1	BioLegend
CD8	APC/eFlour	301014	RPA-T8	BioLegend
Zombie – NIR™ Fixable Viability Staining KIT	APCCy7	423106	-	BioLegend
CD19	APCCy7	302218	HIB19	BioLegend
CD14	APCCy7	367108	63D3	BioLegend

Supplementary Table 2: Influence of immunosuppressive drugs on antibody positivity (univariable logistic regression analyses)

	**Antibody positivity after two vaccine doses (n = 82)**											
**Predictors**	**OR**	p	**OR**	p	**OR**	p	**OR**	p	**OR**	p	**OR**	p
Everolimus use	1.90 (0.68 – 5.93)	0.238										
Cyclosporine A use			0.69 (0.17 – 2.35)	0.570								
Mycophenolate use					0.36 (0.14 – 0.92)	**0.037**						
Prednisolone use							0.14 (0.05 – 0.38)	**<0.001**				
Tacrolimus use									2.47 (0.90 – 7.63)	0.093		
Use of any calcineurin inhibitor											3.54 (0.85 – 24.14)	0.120

	**Antibody positivity after three vaccine doses (n = 70)**											
**Predictors**	**OR**	p	**OR**	p	**OR**	p	**OR**	p	**OR**	p	**OR**	p
Everolimus use	3.30 (1.12 – 10.17)	**0.032**										
Cyclosporine A use			1.04 (0.28 – 4.35)	0.954								
Mycophenolate use					0.37 (0.13 – 0.98)	0.051						
Prednisolone use							0.37 (0.13 – 1.01)	0.057				
Tacrolimus use									1.02 (0.36 – 2.82)	0.964		
Use of any calcineurin inhibitor											1.07 (0.29 – 3.65)	0.913

Supplementary Table 3: Tobit linear regression model with log-transformed anti-spike IgG concentrations as the outcome variable

	**Log-transformed anti-SARS-CoV-2-spike IgG concentrations after two vaccine doses (n = 82)**											
**Predictors**	**Estimates**	p	**Estimates**	p	**Estimates**	p	**Estimates**	p	**Estimates**	p	**Estimates**	p
Age at first vaccine dose	-0.07 (-0.12 – -0.02)	**0.007**	-0.07 (-0.12 – -0.02)	**0.009**	-0.07 (-0.11 – -0.02)	**0.008**	0.07 (-0.11 – -0.03)	**0.001**	-0.06 (-0.11 – -0.01)	**0.013**	-0.07 (-0.12 – -0.02)	**0.009**
Male Gender	-1.15 (-2.52 – 0.23)	0.102	-1.13 (-2.52 – 0.25)	0.109	-0.99 (-2.35 – 0.37)	0.152	-1.13 (-2.32 – 0.06)	0.062	-1.07 (-2.47 – 0.34)	0.137	-1.00 (-2.41 – 0.40)	0.161
Everolimus use	1.07 (-0.44 – 2.58)	0.163										
Cyclosporine A use			0.22 (-1.64 – 2.07)	0.820								
Mycophenolate use					-1.28 (-2.58 – 0.03)	0.056						
Prednisolone use							-2.72 (-3.86 – -1.58)	**<0.001**				
Tacrolimus use									0.35 (-1.14 – 1.84)	0.642		
Use of any calcineurin inhibitor											0.82 (-1.11 – 2.75)	0.404

	**Log-transformed anti-SARS-CoV-2-spike IgG concentrations after three vaccine doses (n = 70)**											
**Predictors**	**Estimates**	p	**Estimates**	p	**Estimates**	p	**Estimates**	p	**Estimates**	p	**Estimates**	p
Age at first vaccine dose	-0.05 (-0.11 – 0.02)	0.151	-0.07 (-0.13 – -0.00)	**0.046**	-0.05 (-0.11 – 0.01)	0.118	-0.07 (-0.13 – -0.01)	**0.023**	-0.07 (-0.13 – -0.00)	**0.038**	-0.06 (-0.12 – 0.00)	0.059
Male Gender	-0.42 (-2.12 – 1.29)	0.634	-0.50 (-2.25 – 1.26)	0.580	-0.22 (-2.00 – 1.56)	0.806	-0.52 (-2.17 – 1.14)	0.539	-0.66 (-2.41 – 1.09)	0.458	-0.68 (-2.48 – 1.11)	0.456
Everolimus use	1.98 (0.11 – 3.85)	**0.038**										
Cyclosporine A use			0.79 (-1.54 – 3.11)	0.508								
Mycophenolate use					-1.26 (-2.94 – 0.42)	0.141						
Prednisolone use							-1.84 (-3.37 – -0.31)	**0.018**				
Tacrolimus use									-0.83 (-2.59 – 0.93)	0.357		
Use of any calcineurin inhibitor											-0.53 (-2.62 – 1.56)	0.619

Supplementary Table 4: Influence of number of immunosuppressive drugs used: Logistic regression analysis of two (reference) vs. three drugs

	After two vaccine doses (n = 80)		After three vaccine doses (n = 68)	
**Predictors**	**OR (95% CI)**	**p**	**OR (95% CI)**	**p**
Age	0.95 (0.91 – 0.99)	**0.012**	0.98 (0.94 – 1.02)	0.351
Male Sex	0.40 (0.12 – 1.27)	0.124	0.69 (0.20 – 2.20)	0.534
Three immunosuppressive drugs	0.09 (0.02 – 0.28)	**<0.001**	0.37 (0.12 – 1.06)	0.071
